# Case report: Immune modulation after PD-1 inhibitor therapy in a patient with extranodal NK/T-cell lymphoma secondary to chronic active Epstein-Barr virus disease unveiled by single-cell transcriptomics

**DOI:** 10.3389/fimmu.2023.1172307

**Published:** 2023-04-17

**Authors:** Yao Wang, Minan Zhang, Qingfeng Xue, Huan Zhou, Jie Chen, Hong Wang, Yaping Zhang, Wenyu Shi

**Affiliations:** ^1^ Department of Hematology, Affiliated Hospital of Nantong University, Nantong, China; ^2^ Department of Oncology, Affiliated Hospital of Nantong University, Nantong, China

**Keywords:** chronic active Epstein-Barr virus disease, extranodal NK/T-cell lymphoma, PD-1 inhibitor, single-cell transcriptomics, hemophagocytic lymphohistiocytosis

## Abstract

Chronic active Epstein-Barr virus disease (CAEBV) is a systemic lymphoproliferative disorder that is closely linked to Epstein-Barr virus (EBV) infection. The clinical course and severity of CAEBV can vary, and in some cases, it can progress to overt lymphoma, which is characterized by extranodal natural killer/T-cell lymphoma (ENKTL) and has a poor clinical outcome. Although anti-programmed cell death protein-1 (PD-1) therapy has shown effectiveness in some patients with EBV-associated disease, it has been less successful in others, and the exact mechanism of action of PD-1 inhibitor therapy in these diseases remains unclear. In this report, we describe a patient who was diagnosed with ENKTL secondary to CAEBV and experienced rapid disease progression accompanied by hyperinflammation after receiving PD-1 inhibitor therapy. Single-cell RNA sequencing revealed a significant increase in the patient’s lymphocyte count, especially in natural killer cells, with increased activity following PD-1 inhibitor therapy. This case raises questions about the efficacy and safety of PD-1 inhibitor therapy in patients with EBV-associated diseases.

## 1 Introduction

Chronic active Epstein-Barr virus disease (CAEBV) is a systemic disorder that typically presents with symptoms such as fever, persistent hepatitis, hepatosplenomegaly, and lymphadenopathy lasting for at least three months. CAEBV is closely associated with Epstein-Barr virus (EBV) infection and is characterized by proliferating EBV-positive T-cells or natural killer (NK) cells (1). The clinical course and severity of CAEBV can vary, depending on factors such as the age of onset, individual immune response, EBV load, and the predominant infected cell type. While some patients can survive for many years without progression, adults with EBV-infected T-cells may have a shorter survival time. Approximate 16% of cases progress to overt lymphoma or leukemia, such as extranodal NK/T-cell lymphoma (ENKTL), which have a poor prognosis (1, 2). Additionally, about 24% of patients may develop life-threatening hemophagocytic lymphohistiocytosis (HLH) as the disease progresses (3).

While various treatments have been used to alleviate symptoms of CAEBV, such as immunomodulatory therapy, antiviral therapy, EBV-specific T-cell therapy, and chemotherapy, hematopoietic stem cell transplantation is currently the only potentially curative option (4). Patients with ENKTL have responded well to L-asparaginase-containing chemotherapy and radiotherapy (5); however, there is no standardized therapeutic regimen for ENKTL secondary to CAEBV (2, 6). Anti-programmed cell death protein-1 (PD-1) therapy, an immune checkpoint inhibitor, has shown effectiveness in some patients with EBV-associated NK/T-cell lymphoma and EBV-associated HLH (7, 8), but not in all cases. In this report, we describe a patient with ENKTL secondary to CAEBV who developed HLH and experienced rapid disease progression after receiving anti-PD-1 therapy. We investigated the patient’s immune status by performing single-cell RNA sequencing (scRNA-seq) before and after anti-PD-1 therapy to predict possible disease progression trajectories.

## 2 Case description

**Figure 1:**
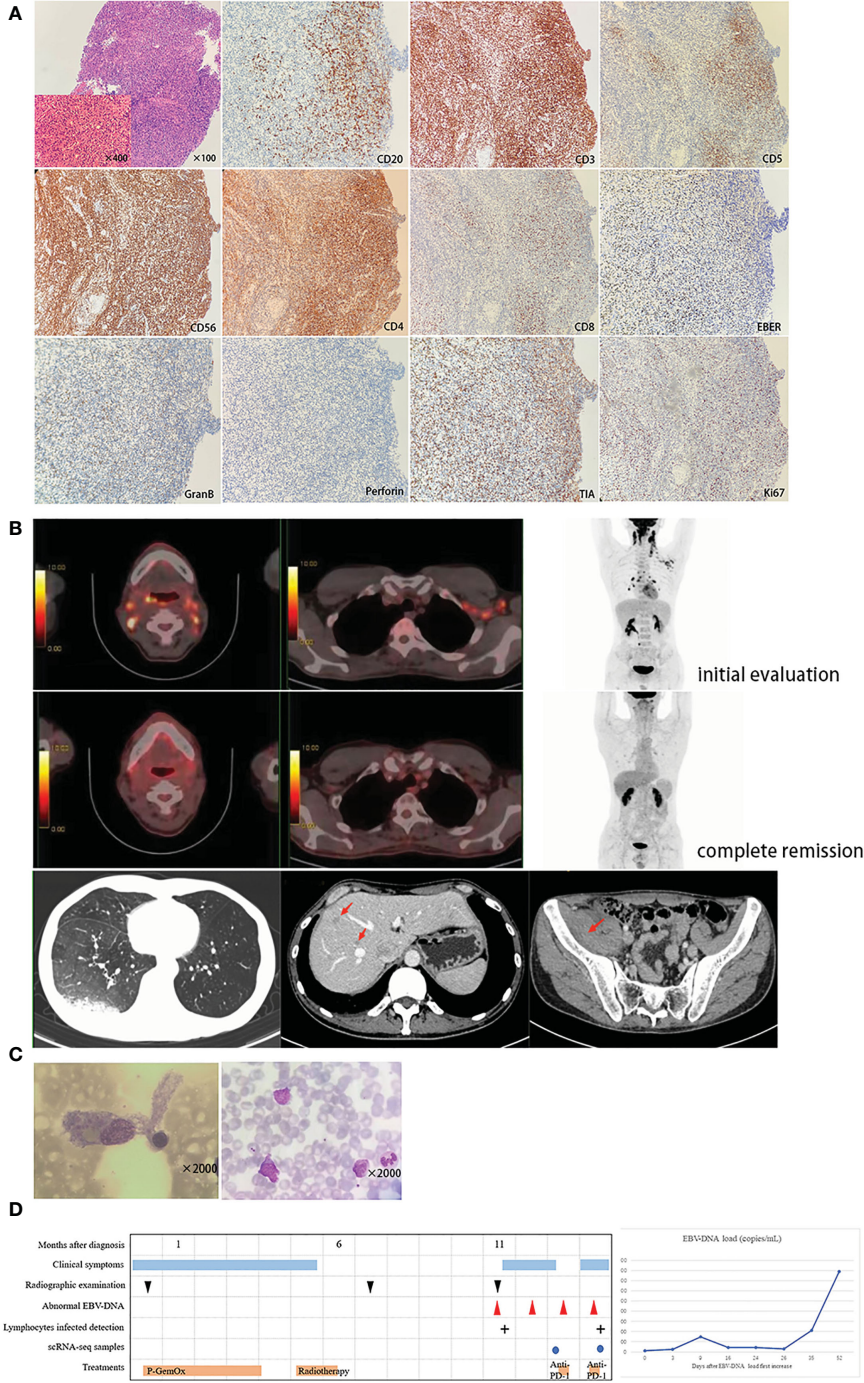
**(A)** Immunohistochemical staining of hematoxylin-eosin staining (H&E) of nasopharyngeal mucosa were presented with infiltrate of atypical lymphoid cells in the setting of coagulative necrosis and immunohistochemistry showed these neoplastic cells were positive for CD56, CD3, TIA, GranB, Perforin, Ki67 (50%+), CD4 (majority of cells), CD8 (minority of cells) and EBV-encoded RNA, and negative for CD20 and CD5. **(B)** Radiographic examinations of different disease stage: Up: Hypermetabolism lesions were seen in nasopharyngeal mucosa and cervical and thoracic lymph node on PET image when initially diagnosed. Middle: PET/CT scanning showed complete remission after chemotherapy and nasal radiation. Down: Enhanced CT scanning showed exudative lesion of the right lower lobe of the lung, multiple low-density patches in liver and obvious edema of the right-side iliopsoas muscle. **(C)** Bone marrow smear showed abnormal phagocytes engulfing normal blood cells (Left) and a blood smear showed heterotypic lymphocytes (Right). **(D)** Clinical course (Left) and increased EBV-DNA load (Right) of the patient were presented.

A 48-year-old man was admitted to our hospital in August 2020 with a one-month history of persistent pharyngodynia, fever, and weight loss that had not responded to antibiotics. Bilateral thickening of the nasal mucosa and yellowish-white plaques in the posterior wall of the nasopharynx were observed during nasopharyngoscopy, which were later diagnosed as nasopharyngeal neoplasms. The patient had a six-year history of untreated chronic inflammation of the nasopharyngeal mucosa with lymphadenosis, as seen in **Figure S1A and 1B**. Pathologic examination of the neoplasms revealed hyperplastic lymphoid tissue with necrosis (**Figure 1A**). Immunohistochemistry showed that the neoplastic cells were positive for CD56, CD3, TIA, GranB, Perforin, Ki67 (50%+), CD4 (majority of cells), CD8 (minority of cells), and EBV-encoded RNA, but negative for CD20 and CD5 (**Figure 1A**), supporting a pathological diagnosis of ENKTL, nasal type. The whole-blood EBV-DNA test was negative, and flow cytometry showed a low CD4/CD8 ratio. Full-body positron emission tomography/computed tomography (PET/CT) revealed hypermetabolism in the thickened tissue of the nasopharynx (maximum standardized uptake value 25.1), areas of tumor infiltration in the nasal cavity, oropharynx, and tonsillar tissue, as well as multiple enlarged cervical, hilar, mediastinal, and axillary lymph nodes of varying sizes (**Figure 1B**). Based on the patient’s symptoms, immunohistochemistry, and PET/CT findings, we diagnosed ENKTL, nasal type (stage II, group B), which was considered low risk according to the PINK-E prognostic model.

After six courses of P-GemOx treatment and nasal radiotherapy with 52 Gy administered in 26 fractions, the patient achieved complete remission with decreased metabolism in the areas of tumor infiltration (**Figure 1B**). Follow-up was planned (**Figure 1B**). Although the patient had no relevant clinical signs, his EBV-DNA load increased to 2680 copies/mL after three months. A contrast-enhanced CT revealed an exudative lesion on the right lower lobe of the lung, multiple low-density patches in the liver, and edema of the iliopsoas muscle on the right side, indicating either infection or tumor recurrence (**Figure 1B**). Since needle biopsy was not possible due to the deep location, the patient was scheduled for reexamination after antimicrobial treatment. However, a week later, the patient developed a fever of 38°C and jaundice. Laboratory results showed high hepatic transaminase, pancytopenia, hypertriglyceridemia, hypofibrinogenemia, and elevated serum ferritin. Splenomegaly was observed, suggesting a diagnosis of HLH. The patient’s bone marrow smear revealed an abundance of abnormal phagocytes engulfing normal blood cells, and a blood smear showed heterotypic lymphocytes (**Figure 1C**). Flow cytometry of bone marrow showed that about 3.9% of cells had the NK phenotype, and were strongly positive for CD56, CD7, CD45, CD16 (including a proportion of CD16dim), positive for CD2, and negative for CD3, CD5, CD57, CD4, and CD8 (**Figure S2A**). Flow cytometry of peripheral blood cells showed that the proportion of CD56-positive lymphocytes had increased to 67.7% with abnormal expression of killer inhibitory receptors, no CD158a, CD158e or CD158i, and low CD158b expression (1.5%) (**Figure S2C**). EBV-DNA remained at a high level (up to 29,700 copies/mL), and high-throughput DNA sequencing identified only EBV as a pathogenic microorganism. Cell sorting by quantitative real-time polymerase chain reaction showed that the lymphocytes infected with EBV were CD4-positive T-cells and CD56-positive cells. Treatment with methylprednisolone and etoposide was started and partially improved the patient’s symptoms. However, contrast-enhanced CT showed that the lesions had decreased in size, but flow cytometry revealed that 7.3% of the NK cells in bone marrow were still abnormal (**Figure S2B**). The EBV-DNA level remained persistently high. An intravenous PD-1 inhibitor (penpulimab, 200 mg every two weeks) was administered, which significantly improved the patient’s clinical symptoms and abnormal laboratory parameters. However, the disease subsequently progressed rapidly, with persistent pancytopenia and deteriorating liver function tests. After anti-PD-1 therapy, cell sorting detected that the lymphocytes infected with EBV were CD4-positive T-cells, CD8-positive T-cells, B-cells, and CD56-positive cells. Unfortunately, the patient died a month after receiving anti-PD-1 therapy (**Figure 1D**).

ScRNA-seq of peripheral blood mononuclear cell (PBMC) samples obtained before and after PD-1 inhibitor therapy was performed by Singleron Biotechnologies. The methods were shown in the supplements and main results were shown as follows:

### 2.1 ScRNA-seq identified major immune cell populations in peripheral blood

**Figure 2:**
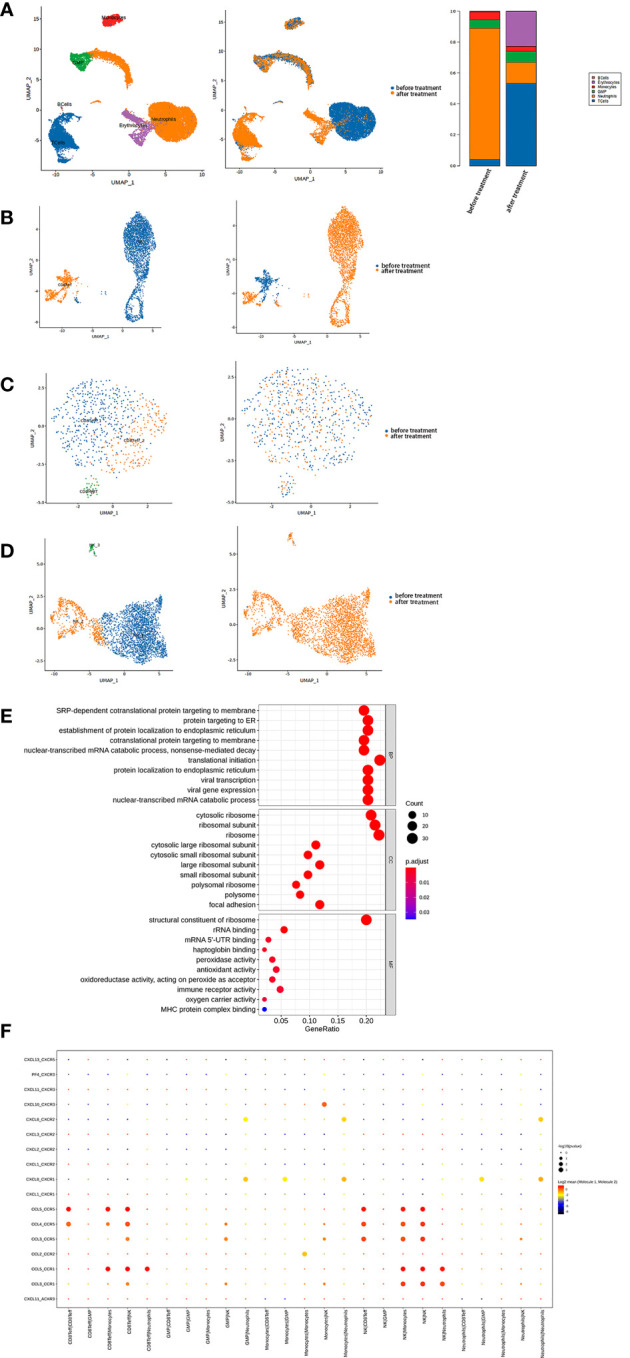
**(A)** Single-cell transcriptome profiling of PBMCs from the patient showed that two-dimensional UMAP visualization of PBMCs resulted in 6 clusters (Left) and were classified based on different samples (Middle). Finally, the fractions of all cell clusters were presented before and after PD-1 therapy (Right). **(B)** T-cells were further subdivided into 2 clusters (Left) and classified based on different samples (Right). **(C)** CD8+ effector T-cells were further subdivided into three clusters (Left) and classified based on different samples (Right). **(D)** NK cells were further subdivided into three clusters (Left) and classified based on different samples (Right). **(E)** Top 10 biological processes by GO gene set enrichment analysis in NK cells were shown in bubble plot according to gene ratio.

We analyzed 11,666 cells collected before and 6,284 cells collected after anti-PD-1 therapy. Using unbiased clustering, we identified six distinct cell types, including T-cells, B-cells, neutrophils, monocytes, granulocyte-monocyte progenitor cells, and erythrocytes (**Figure 2A**). We further classified these cells according to their source to compare cell type changes (**Figure 2A**). Notably, we observed a significant increase in T-cells and a marked decrease in neutrophils after anti-PD-1 therapy. To gain insight into the underlying mechanisms, we performed detailed analyses of the key cell populations, including subclustering of cells, pseudo-time analysis, gene set enrichment analysis, and pathway enrichment analysis.

### 2.2 Depletion of T-cells and enhanced activity in NK cells after anti-PD-1 therapy

Among the immune cell populations, T-cells were found to have the most significant impact on the patient’s condition. Further subclustering identified two main clusters: CD8+ effector T-cells and NK cells (**Figure 2B**).

The CD8+ effector T-cells were further divided into three clusters (**Figure 2C**). Although there was an overall reduction in the CD8+ effector T-cell count after anti-PD-1 therapy, the constitution of each cluster was similar (**Figure S3A**). Remarkably, these three clusters showed enhanced cytotoxicity according to type of UCell signature score (**Figure S3C**). We observed upregulation of the type I interferon signaling pathway and a marked response to type I interferon by the Gene Ontology (GO) analysis, indicating antiviral and antitumor activity (**Figure S3D**).

However, there was a marked increase in NK cells after treatment. We found the pathways of SRP-dependent cotranslational protein targeted to the membrane, protein targeted to endoplasmic reticulum, and establishment of protein localization to endoplasmic reticulum were significantly upregulated in NK cells, indicating increased protein production (**Figure 2E**). Furthermore, NK cells secreted mainly pro-inflammatory chemokines, including CCL3, CCL4, and CCL5. CellPhoneDB results showed that the receptors for these chemokines on monocytes, neutrophils, and CD8+ effector T-cells were responsible for the interaction between these cells after anti-PD-1 therapy (**Figure 2F**).

The NK cells were further divided into three regional clusters (**Figure 2D**, **Figure S3B**). NK_1, which appeared to be increased after therapy, was found at the start of differentiation from the pseudo-time analysis result (**Figure S3E**) with an upregulated type I interferon signaling pathway and an enhanced response to type I interferon. NK_2 was upregulated in pathways related to production of ribosomes (e.g. biogenesis of the ribonucleoprotein complex and ribosome). The pseudo-time analysis of NK_3 was different from that of NK_2. In NK_3, the upregulated pathways were primarily associated with an enhanced neutrophil-associated immune response, which was in contrast with other NK cell clusters (**Figure S3F**). Overall, our results showed that the immune response was enhanced, especially in NK cells, after anti-PD-1 therapy.

### 2.3 Identification of myeloid cells with abnormal phenotypes

**Figure 3:**
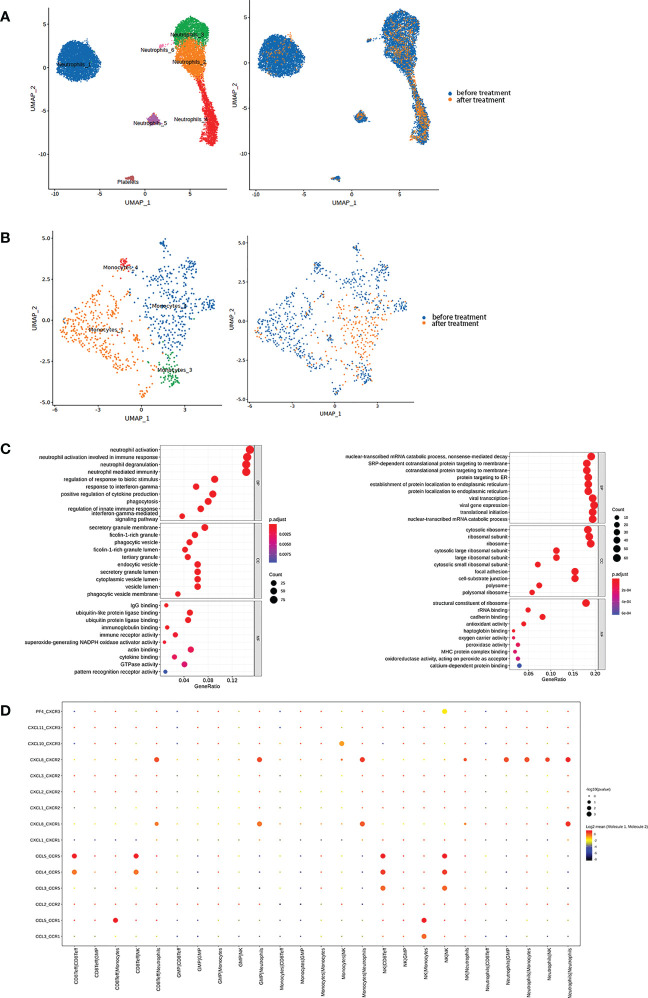
**(A)** Neutrophils were further subdivided into six clusters (Left) and classified based on different samples (Right). **(B)** Monocytes were further subdivided into three main clusters (Left) and classified based on different samples (Right). **(C)** Top 10 biological processes by GO gene set enrichment analysis before (Left) and after (Right) therapy in neutrophils were shown in bubble plot according to gene ratio. **(D)** The interaction between each cluster by cytokines in the sample before therapy was presented.

We also found a marked decrease in myeloid cells, including neutrophils and monocytes, after anti-PD-1 therapy. Normally, neutrophils would be removed during process of single-cell sequencing samples. However, we continued to find low-density neutrophils (LDNs) in our samples, especially after onset of HLH before anti-PD-1 therapy. The proportion of neutrophils in the sample was high and was further classified into six clusters (**Figure 3A**, **Figure S4A**). According to the GO gene set enrichment analysis results, we found that pathways involved in enhanced neutrophil functions were the main biological processes in all neutrophil clusters before anti-PD-1 therapy, while pathways associated with protein production were upregulated after therapy and corresponded to NK cells (**Figure 3C**). Neutrophils_1, accounting for the largest percentage before therapy, showed high expression of genes (e.g. G0S2, CXCL8, CLEC2B, and CXCR2) which have a role in the biological function of neutrophils and is a cluster similar to normal neutrophils and participate in the inflammation process. In neutrophils_2, genes related to the cell cycle and cell proliferation and differentiation (e.g. LGALS1, CA4, and S100A4) were highly expressed and tended to represent the stage before maturation of neutrophils. Neutrophils_3 was a cluster with high expression of HLA genes (e.g. HLA-DRA, HLA-DMA, HLA-DMB, and CD74) and genes associated with antiviral therapeutics (e.g. IFIT1, HERC5, MX1, RSAD2, and ISG15). These neutrophils can function as antigen-presenting cells and have antiviral activity. Neutrophils_4, with the highest percentage of neutrophils after PD-1 therapy, was enriched with genes that have an antimicrobial function (e.g. LTF, CAMP, RETN, and PGLYRP1). This cluster was also upregulated in pathways involved in the response to interferon-γ and viruses and in the type I interferon signaling pathway and might be associated with enhanced activity of NK cells (**Figure S4E**). In neutrophils_5, high expression of immunoglobulin genes (e.g. IGKC, IGLL3P, IGHE, and IGHM) and antimicrobial genes (e.g. ELANE, DEFA4, CCL5, and GZMA) indicated that these neutrophils also had high activity. Finally, neutrophils_6, contained the smallest proportion, was highlyexpressed genes related to mitochondrial biology, and this cluster may be involved in active proliferation of these cells (**Figure S4C**). CellPhoneDB results indicated that neutrophils maintain a close relationship with other immune cells by secreting and receiving CXCL8 and could be closely associated with an inflammatory response such as HLH (**Figure 3D**).

Furthermore, monocytes were activated before anti-PD-1 therapy and partitioned into three main clusters (**Figure 3B**, **Figure S4B**). Monocytes_1 expressed markers such as PLAC8 and CLU (**Figure S4D**), which have been identified to be a unique monocytic subset in emergency myelopoiesis (9). We thought that this cluster was intimately connected with the onset of HLH. Like the NK_3 cluster, Monocytes_2 and Monocytes_3 upregulated pathways for neutrophil-mediated immunity (**Figure S4F**). CellPhoneDB results indicated that monocytes secreting CXCL8 as an inflammatory mediator interacted with neutrophils *via* CXCR1 and CXCR2 (**Figure 3D**). These findings suggested that the patient’s HLH might be closely related to the function of myeloid cells, especially neutrophils.

## 3 Discussion

There are eight diseases characterized by proliferations of EBV-positive T-cells and NK cells, which include CAEBV, ENKTL and aggressive NK cell leukemia. These diseases share several features, such as a cytotoxic phenotype, disproportionately high CD56 positivity, a consistent association with EBV, a tendency to involve extranodal sites, and aggressive clinical behavior (1). In this report, we present a case of a patient diagnosed with ENKTL secondary to CAEBV. Although the patient showed the initial positive response after anti-PD-1 therapy, there was rapid disease progression during follow-up.

CAEBV is a progressive disease that causes persistent inflammation and the clonal proliferation of infected T-cells or natural killer cells (1). In this case, the patient had a history of chronic inflammation of the nasopharyngeal mucosa and lymphadenosis. Immunohistochemistry results showed that most of the cells were CD4-positive, and cell sorting confirmed that the infected cells were CD4-positive T-cells and CD56-positive cells. As the disease progressed, the levels of EBV-DNA increased rapidly, which is typical of CAEBV. Based on these findings, the patient was diagnosed with ENKTL secondary to CAEBV (10, 11). ENKTL is a type of lymphoma that responds well to L-asparaginase-containing chemotherapy and radiotherapy (5). However, CAEBV is refractory to many treatments used to alleviate symptoms (4, 12). The patient’s rapidly disseminating and deteriorating disease, along with their history of nasal symptoms, increasing EBV-DNA, and the presence of EBV-infected CD4-positive T-cells and CD56-positive cells, strongly suggested a diagnosis of ENKTL secondary to CAEBV.

The complications that arose in this case raise questions about the effectiveness of PD-1 inhibitor therapy in hematological malignancies. Recently, a PD-1 immune checkpoint inhibitor has demonstrated promise in the treatment of hematological malignancies by restoring the activity of effector T-cells. EBV infection can induce expression of PD-L1, leading to exhaustion of T-cells and causing lymphoma, which theoretically supports use of PD-1 inhibitor therapy. A multi-omics study performed on 128 biopsy samples from patients with ENKTL identified three distinct molecular subtypes, TSIM, MB, and HEA, and found overexpression of PD-L1 in the TSIM subtype (13). In a clinical study that included seven patients with refractory/relapsed ENKTL, all achieved a 100% objective response rate and 71.4% achieved a complete response after seven cycles of PD-1 inhibitor therapy (7). In another report, PD-1 inhibitor therapy was used successfully as a salvage treatment in a post-transplant patient, in whom EBV was completely eradicated (14). In a retrospective analysis of seven patients with EBV-associated HLH, five (71.4%) achieved a sustained clinical complete response (8).

Nevertheless, only a small number of patients have received this treatment so far. Therefore, no definite conclusions can be reached regarding its efficacy in EBV-associated diseases. In view of the lack of a curative effect of anti-PD-1 therapy in our patient, we isolated PBMCs before and after treatment and profiled them to observe the response to treatment. Comparison of his PMBCs before and after therapy showed marked decreases in neutrophils and monocytes and a significant increase in T-cell lines, especially NK cells. In the setting of HLH, cytotoxic T lymphocytes (CTLs) and NK cells could not clear the infected cells or tumor cells, and the activity of regulatory T-cells, which can inhibit CTLs, is impaired resulting in continuous proliferation of CTLs and NK cells. Finally, overactivated CTLs release interferon-γ, which activates macrophages and causes hyperinflammation (15). We found that although the symptoms in our patient were similar to those of a cytokine storm, our sequencing results indicated his symptoms had a stronger relationship with myeloid cells before treatment and more likely reflected hyperactive cytotoxic NK cells after treatment. We identified an abnormal increase in NK cells with enhanced cytotoxicity and a simultaneous depletion of CD8+ T-cells. PD-1 expression is mainly promoted by activated T-cells, and those T-cells could inhibit the overexpression of PD-1 in turn. (16, 17). In the tumor microenvironment, cancer cells could allow themselves to escape immune surveillance by the interaction between PD-1 and PD-L1 (16, 18). Antibodies to PD-1 block the pathway, partially restoring T-cell function so that these cells can continue to kill cancer cells. Recent research indicates that NK cells could also be activated by blockade of the PD-1 checkpoint (19). Consistent with this finding, scRNA-seq showed that our patient had a marked increase in NK cells with enhanced secreting and killing capacity after anti-PD-1 therapy. CellPhoneDB results indicated that the main cytokines involved were inflammatory chemokines (CCL3, CCL4, and CCL5) from NK cells. These chemokines belong to the CC subfamily and act primarily on monocytes and lymphocytes. The biological effects were mediated *via* incorporation of CC chemokine receptors, which are members of the G protein-coupled receptor superfamily, on the cell surface. Previous studies have demonstrated that a combination of CCL3 or CCL5 and CCR5 might cause severe inflammation and damage in acute pancreatitis, inflammatory bowel disease, and lung tissue (20–23). Thus, NK cells have a close relationship with the onset of a cytokine storm, which might explain why our patient had symptoms similar to HLH after therapy.

EBV-associated HLH was previously believed to be caused by a cytokine storm triggered by macrophagocytes (15). In our patient, scRNA-seq showed that C-X-C motif ligand 8 (CXCL8) was the chemokine with the most interaction between neutrophils, monocytes, and other immune cells by CellPhoneDB. CXCL8 is considered a prototypical chemokine in the CXC family and recruits and activates neutrophils and granulocytes to sites of inflammation. The function of CXCL8 is mainly dependent on its interaction with the specific cell surface G protein-coupled receptors, CXCR1 and CXCR2. The CXCL8-CXCR1/2 pathways activate the major effector molecules, PLC and PI3K, inducing phosphorylation of PKC and Akt, respectively, which reportedly leads to activation of transcription factors involved in survival, angiogenesis, and migration of tumor cells (24). Moreover, a large number of abnormal LDNs were identified by scRNA-seq. A previous study showed that LDNs contained both mature and immature neutrophils, which were generated during tumor progression and might promote progressive disease (25). Although different clusters were identified in our analysis, all LDNs exhibited activated neutrophil functions. The interaction between these LDNs and other immune cells *via* the CXCL8-CXCR1/2 cytokine pathways suggests a possible relationship with the occurrence of HLH before anti-PD-1 therapy. Interestingly, anti-PD-1 therapy reduced the number of tumor-associated LDNs in our patient, even though the outcome was not satisfactory.

In conclusion, our study provides novel insights into the mechanisms underlying CAEBV and other EBV-associated diseases, using scRNA-seq. We found that these diseases can produce similar symptoms, but *via* different mechanisms. Furthermore, our results suggest that PD-1 inhibitor therapy in these patients can cause systemic hyperinflammation, which can be fatal. These findings have important implications for the clinical management of EBV-associated diseases and underscore the need for further research to better understand the pathogenesis of these conditions and the risks associated with different treatment options.

## Data availability statement

The data presented in the study are deposited in the NCBI Gene Expression Omnibus (GEO) repository, accession number GSE228279.

## Ethics statement

Written informed consent was obtained from the individual(s) for the publication of any potentially identifiable images or data included in this article.

## Author contributions

WS and YZ contributed to conception and design of the study. YW, MZ, and QX performed the statistical analysis and wrote the manuscript draft. HZ, JC, and HW collected the clinical data. YW, MZ, QX, HZ, JC, HW, YZ, and WS revised the manuscript. All authors contributed to the article and approved the submitted version.
