# Supplementary material for: Case report: Immune modulation after PD-1 inhibitor therapy in a patient with extranodal NK/T-cell lymphoma secondary to chronic active Epstein-Barr virus disease unveiled by single-cell transcriptomics
Source: Front Immunol. 2023 Apr 17;14:1172307. doi: 10.3389/fimmu.2023.1172307 (PMC10149821; doi:10.3389/fimmu.2023.1172307)
Supplement: Supplementary file 1 [file DataSheet_1.pdf]

Supplementary Material

Case Report: Immune Modulation after PD-1 Inhibitor Therapy in a Patient with Extranodal NK/T-cell Lymphoma Secondary to Chronic Active Epstein-Barr Virus Disease Unveiled by Single-Cell Transcriptomics

Yao Wang[†], Minan Zhang[†], Qingfeng Xue, Huan Zhou, Jie Chen, Hong Wang, Yaping Zhang*, Wenyu Shi*

*** Correspondence:** Wenyu Shi: shiwenyu@hotmail.com; Yaping Zhang: zzyaping@163.com

1 Supplementary Data

The methods of scRNA-seq were shown as follows:

Tissue dissociation and preparation

PBMCs were fractionated by density gradient centrifugation using Ficoll-Paque Plus medium (GE Healthcare, Chicago, IL, USA) and then rinsed in phosphate-buffered saline (PBS) without Ca^{2+} and Mg^{2+}. Red blood cells were depleted by GEXSCOPE® red blood cell lysis buffer (Singleron) for 10 min at 25°C. After centrifugation at 500 ×g for 5 min and resuspension in PBS, the supernatant of the centrifuged blood samples was discarded after centrifugation at 400 ×g for 5 min at 4°C. The PBMCs were then centrifuged at 400 ×g for 10 min at 4°C and prepared without red blood cells. After discarding the supernatant, the PBMCs were resuspended in PBS to obtain single-cell suspensions. The final preparatory step was microscopic evaluation of cell viability using Trypan Blue staining.

Reverse transcription, amplification, and library construction

Single cells were resuspended in PBS (2×10^5 cells/mL) and loaded onto a microchip using a Singleron Matrix® Single Cell Processing System. After collecting Barcoding Beads, the captured mRNA was reverse-transcribed and the resulting cDNA was amplified. The cDNA was then fragmented and processed for sequencing using the adapter ligation method. We prepared scRNA-seq libraries based on the GEXSCOPE Single Cell RNA Library Kit protocol (Singleron). Each library was diluted to 4 nM, pooled for sequencing, and pair - ended reads with a length of 150 bp were generated using a NovaSeq 6000 system (Illumina, San Diego, CA, USA).

Primary analysis of raw read data

Gene expression matrices were generated from the raw scRNA-seq data using the CeleScope v1.4.0 pipeline (https://github.com/singleron-RD/CeleScope). First, the original data were trimmed using CeleScope to remove the adapter sequences, and the poly-A tails and low-quality reads were filtered by Cutadapt v1.17. After extracting cell barcodes and UMI, the data were mapped using STAR v2.6.1a to the human reference genome (version GRCh38). Individual cell UMI and gene counts

were obtained using featurets v2.0.1. The expression matrix files were collected for follow-up analyses.

Quality control, dimension reduction, and clustering

We filtered out samples in which the number of genes was lower than 200 or the fraction of mitochondrial gene counts was over 20%. After filtration, 17,950 cells containing an average of 1003 genes and 3328 UMIs in each cell were analyzed. After dimensionality reduction and clustering with Seurat (v3.1.2), we used the NormalizeData and ScaleData functions to normalize and scale all gene expression, and selected the top 2000 genes showing variation using the FindVariableFeatures function for principal component analysis. We then created several clusters of the top 20 principal components using FindClusters. Batch effects were removed using Harmony. Finally, we used the UMAP algorithm to visualize the clustering result in the two-dimensional parameter space.

Analysis of differentially expressed genes

Seurat's FindMarkers function was used to describe differentially expressed genes (DEGs) according to the Wilcoxon likelihood ratio test with default parameters in view of the high recurrence rate. Genes expressed in more than 10% of the cells in a cluster and with an average log(fold change) value greater than 0.25 were selected as DEGs.

Annotation of cell types

Cell types were dictated by canonical markers found in the DEGs in each cluster using the SynEcoSys database. Heatmaps, dot plots, and violin plots with expression of markers were generated by Seurat (v3.1.2) DoHeatmap, DotPlot and Vlnplot were used to identify different clusters. Cells identified as doublets that expressed markers for multiple cell types were removed manually.

Pathway enrichment analysis

Gene Ontology (GO) annotation was analyzed using the "clusterProfiler" R package v4.0.2 and demonstrated potential functions in DEGs. Pathways with an adjusted p-value of < 0.05 were deemed to be significant. GO gene sets containing molecular function, biological process, and cellular component categories were used as the reference.

Trajectory analysis

We used Monocle2 v2.22.0 to reconstruct cell differentiation trajectories, and the cells were sorted according to spatiotemporal differentiation by highly variable genes. FindVariableFeatures and dimensionality reduction were carried out using DDRTree. Finally, the plot_cell_trajectory function was used to visualize the results.

Analysis of cell-cell interactions

According to the interactions understood to occur between cell clusters via ligands and receptors, cell-cell interactions were analyzed by CellPhoneDB v2.1.7. We performed 1000 random permutations in all cluster labels and calculated the average ligand-receptor expression in a null distribution between the related clusters. Each ligand and receptor expressed in the cells had a cut-off threshold according to the mean distribution of expression across all genes. When a p-value < 0.05

and an average log expression > 0.1 was visualized using the circlize v0.4.10 package in R, the interactions between cells were considered positive and significant.

2 Supplementary Figures

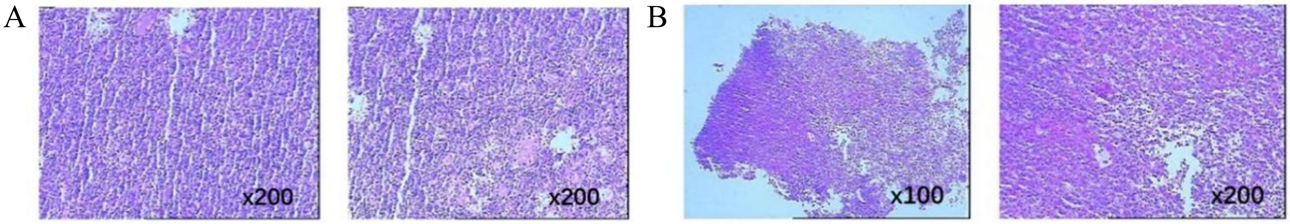

Figure S1. (A) Immunohistochemical staining of H&E of nasopharyngeal mucosa were presented as chronic inflammation with lymphadenosis (2014-03-19). (**B**) Immunohistochemical staining of H&E of nasopharyngeal mucosa were chronic inflammation with localized necrosis and heterocysts infiltrating (2014-04-02).

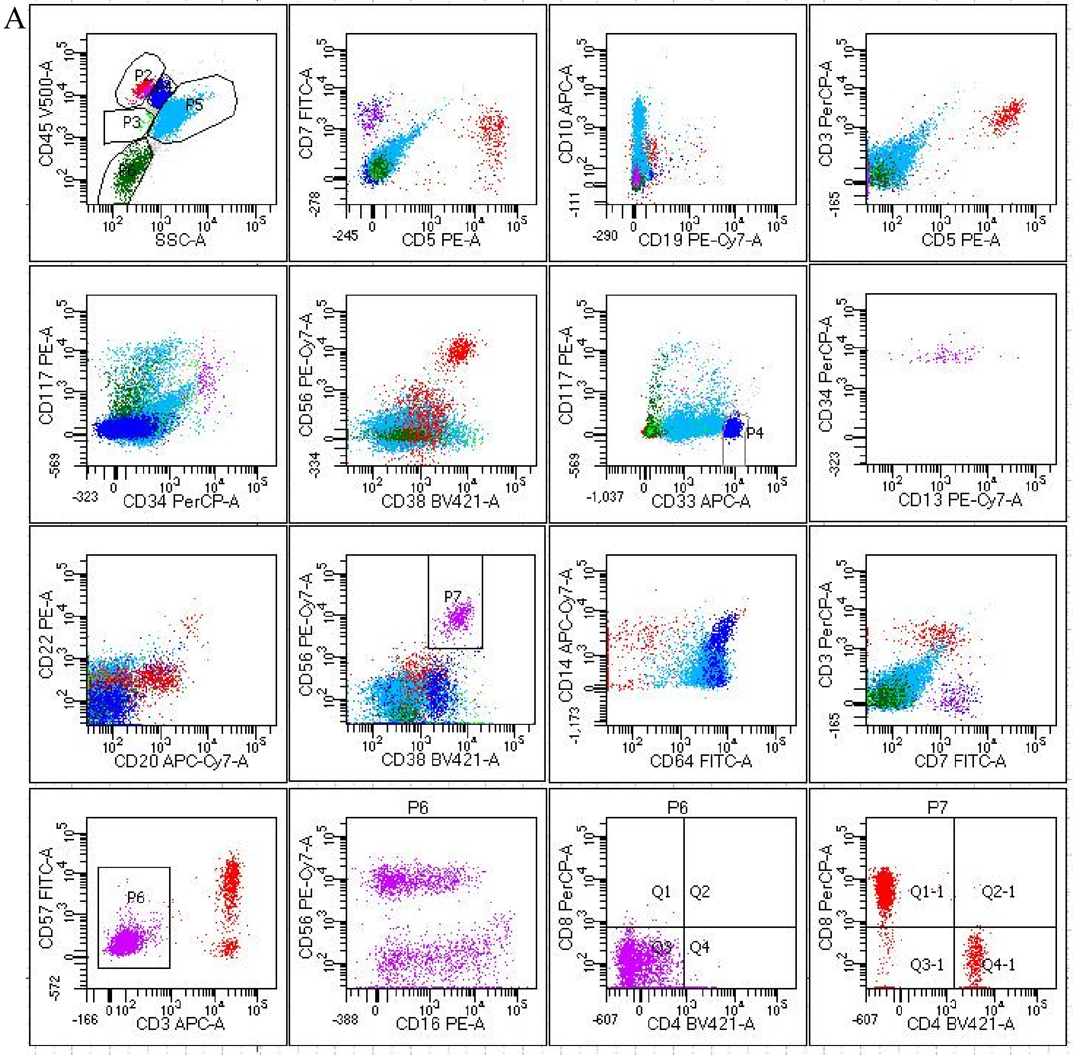

B

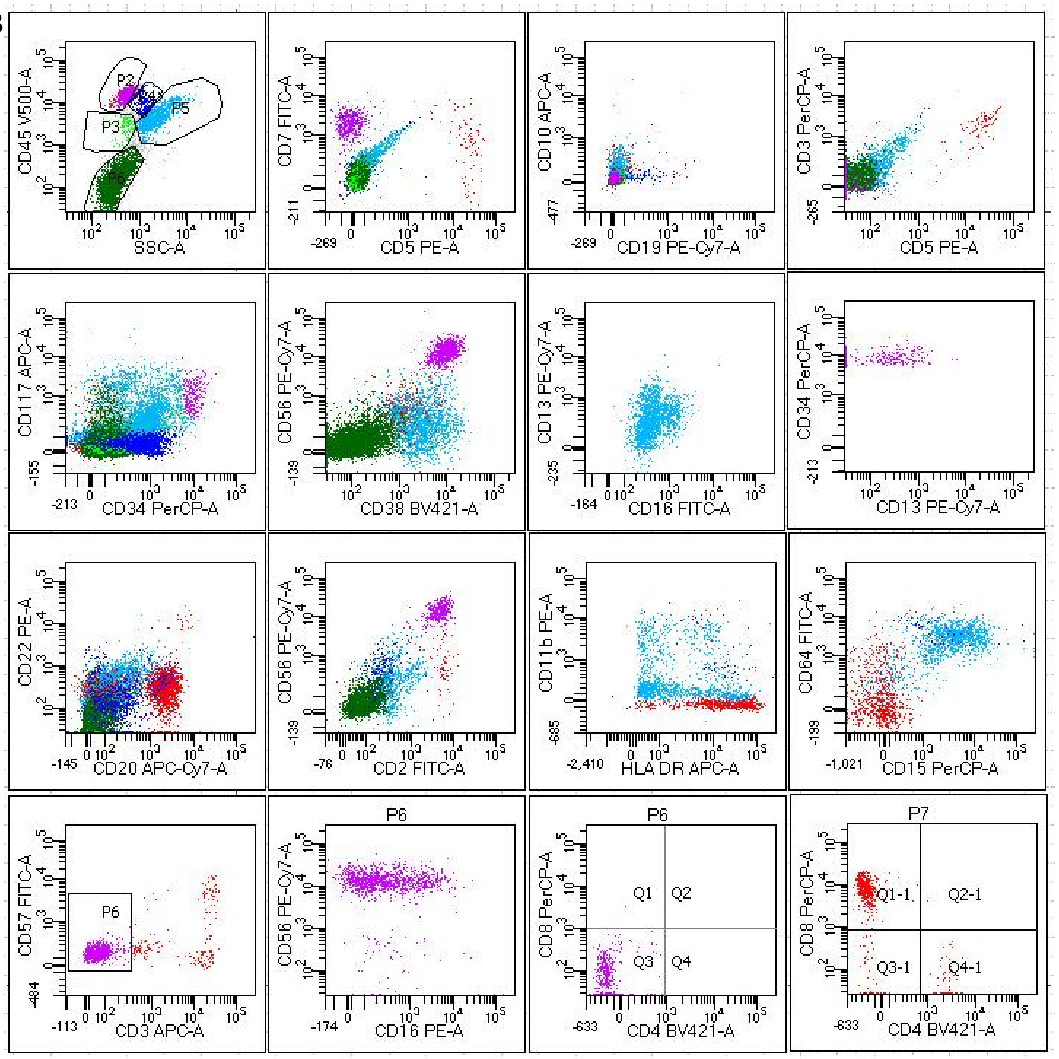

C

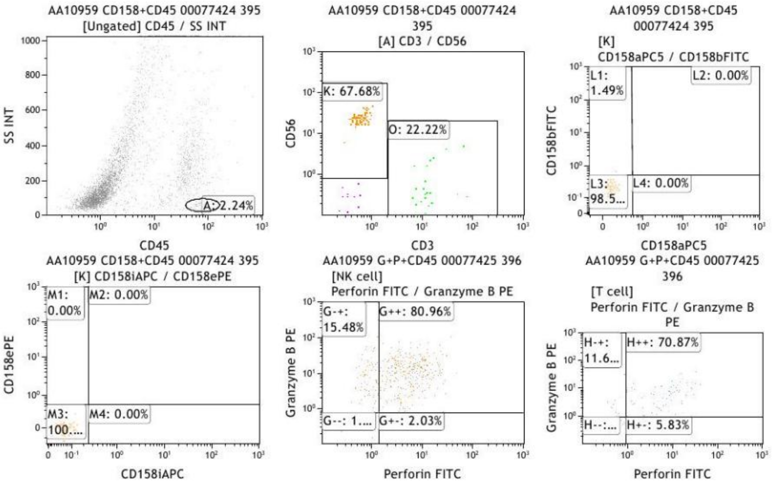

Figure S2. (**A**) Flow cytometry result of NK cells when HLH diagnosed was presented. (**B**) Flow cytometry result of NK cells after treatment with methylprednisolone and etoposide was shown. (**C**) Flow cytometry result showed the expression of killer inhibitory receptors of CD158a, CD158e, CD158i, perforin and granB in NK cells.

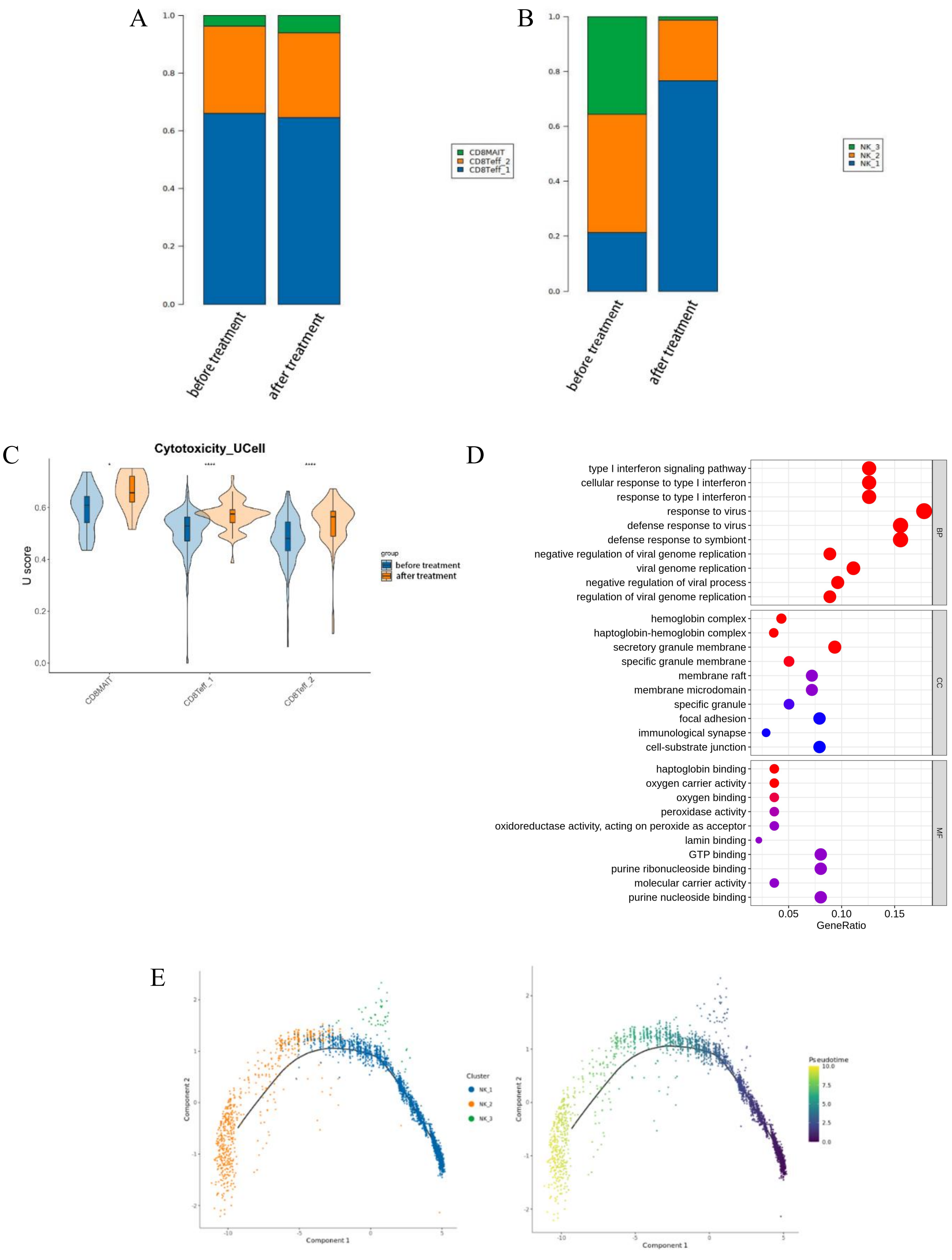
A
CD8MAIT
CD8Teff_2
CD8Teff_1
before treatment
after treatment
B
NK_3
NK_2
NK_1
before treatment
after treatment
C
Cytotoxicity_UCell
U score
group
before treatment
after treatment
CD8MAIT
CD8Teff_1
CD8Teff_2
D
type I interferon signaling pathway
cellular response to type I interferon
response to type I interferon
response to virus
defense response to virus
defense response to symbiont
negative regulation of viral genome replication
viral genome replication
negative regulation of viral process
regulation of viral genome replication
hemoglobin complex
haptoglobin-hemoglobin complex
secretory granule membrane
specific granule membrane
membrane raft
membrane microdomain
specific granule
focal adhesion
immunological synapse
cell-substrate junction
haptoglobin binding
oxygen carrier activity
oxygen binding
peroxidase activity
oxidoreductase activity, acting on peroxide as acceptor
lamin binding
GTP binding
purine ribonucleoside binding
molecular carrier activity
purine nucleoside binding
BP
CC
MF
p.adjust
Count
GeneRatio
E
Cluster
NK_1
NK_2
NK_3
Pseudotime
Component 1
Component 2

F

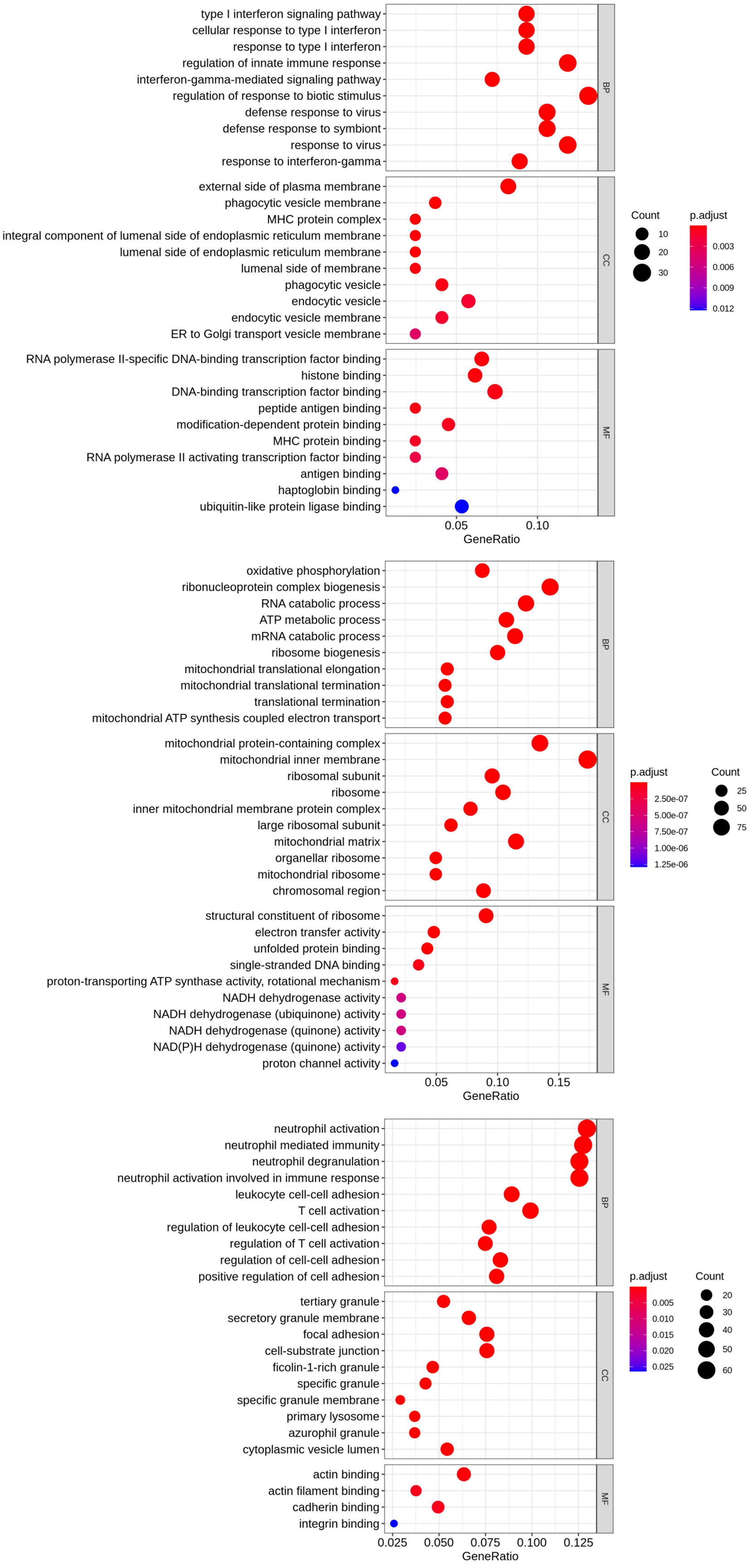
type I interferon signaling pathway
cellular response to type I interferon
response to type I interferon
regulation of innate immune response
interferon-gamma-mediated signaling pathway
regulation of response to biotic stimulus
defense response to virus
defense response to symbiont
response to virus
response to interferon-gamma
external side of plasma membrane
phagocytic vesicle membrane
MHC protein complex
integral component of lumenal side of endoplasmic reticulum membrane
lumenal side of endoplasmic reticulum membrane
lumenal side of membrane
phagocytic vesicle
endocytic vesicle
endocytic vesicle membrane
ER to Golgi transport vesicle membrane
RNA polymerase II-specific DNA-binding transcription factor binding
histone binding
DNA-binding transcription factor binding
peptide antigen binding
modification-dependent protein binding
MHC protein binding
RNA polymerase II activating transcription factor binding
antigen binding
haptoglobin binding
ubiquitin-like protein ligase binding
BP
CC
MF
0.05
0.10
GeneRatio
Count
10
20
30
p.adjust
0.003
0.006
0.009
0.012
oxidative phosphorylation
ribonucleoprotein complex biogenesis
RNA catabolic process
ATP metabolic process
mRNA catabolic process
ribosome biogenesis
mitochondrial translational elongation
mitochondrial translational termination
translational termination
mitochondrial ATP synthesis coupled electron transport
mitochondrial protein-containing complex
mitochondrial inner membrane
ribosomal subunit
ribosome
inner mitochondrial membrane protein complex
large ribosomal subunit
mitochondrial matrix
organellar ribosome
mitochondrial ribosome
chromosomal region
structural constituent of ribosome
electron transfer activity
unfolded protein binding
single-stranded DNA binding
proton-transporting ATP synthase activity, rotational mechanism
NADH dehydrogenase activity
NADH dehydrogenase (ubiquinone) activity
NADH dehydrogenase (quinone) activity
NAD(P)H dehydrogenase (quinone) activity
proton channel activity
BP
CC
MF
0.05
0.10
0.15
GeneRatio
p.adjust
2.50e-07
5.00e-07
7.50e-07
1.00e-06
1.25e-06
Count
25
50
75
neutrophil activation
neutrophil mediated immunity
neutrophil degranulation
neutrophil activation involved in immune response
leukocyte cell-cell adhesion
T cell activation
regulation of leukocyte cell-cell adhesion
regulation of T cell activation
regulation of cell-cell adhesion
positive regulation of cell adhesion
tertiary granule
secretory granule membrane
focal adhesion
cell-substrate junction
ficolin-1-rich granule
specific granule
specific granule membrane
primary lysosome
azurophil granule
cytoplasmic vesicle lumen
actin binding
actin filament binding
cadherin binding
integrin binding
BP
CC
MF
0.025
0.050
0.075
0.100
0.125
GeneRatio
p.adjust
0.005
0.010
0.015
0.020
0.025
Count
20
30
40
50
60

Figure S3. (**A**) The fraction of CD8+ effector T-cells was presented before and after PD-1 inhibitor therapy. (**B**) The fraction of NK cells was presented before and after PD-1 inhibitor therapy. (**C**) CD8+ effector T-cells exhibited enhanced cytotoxicity according to cell type signature score by Ucell. (**D**) Top 10 biological processes by GO gene set enrichment analysis in CD8+ effector-T cells were shown in bubble plot according to gene ratio. (**E**) The pseudo-time analysis result of NK cells was shown. (**F**) Top 10 biological processes by GO gene set enrichment analysis in NK_1 (Up), NK_2 (Middle) and NK_3 (Down) clusters were shown in bubble plot according to gene ratio.

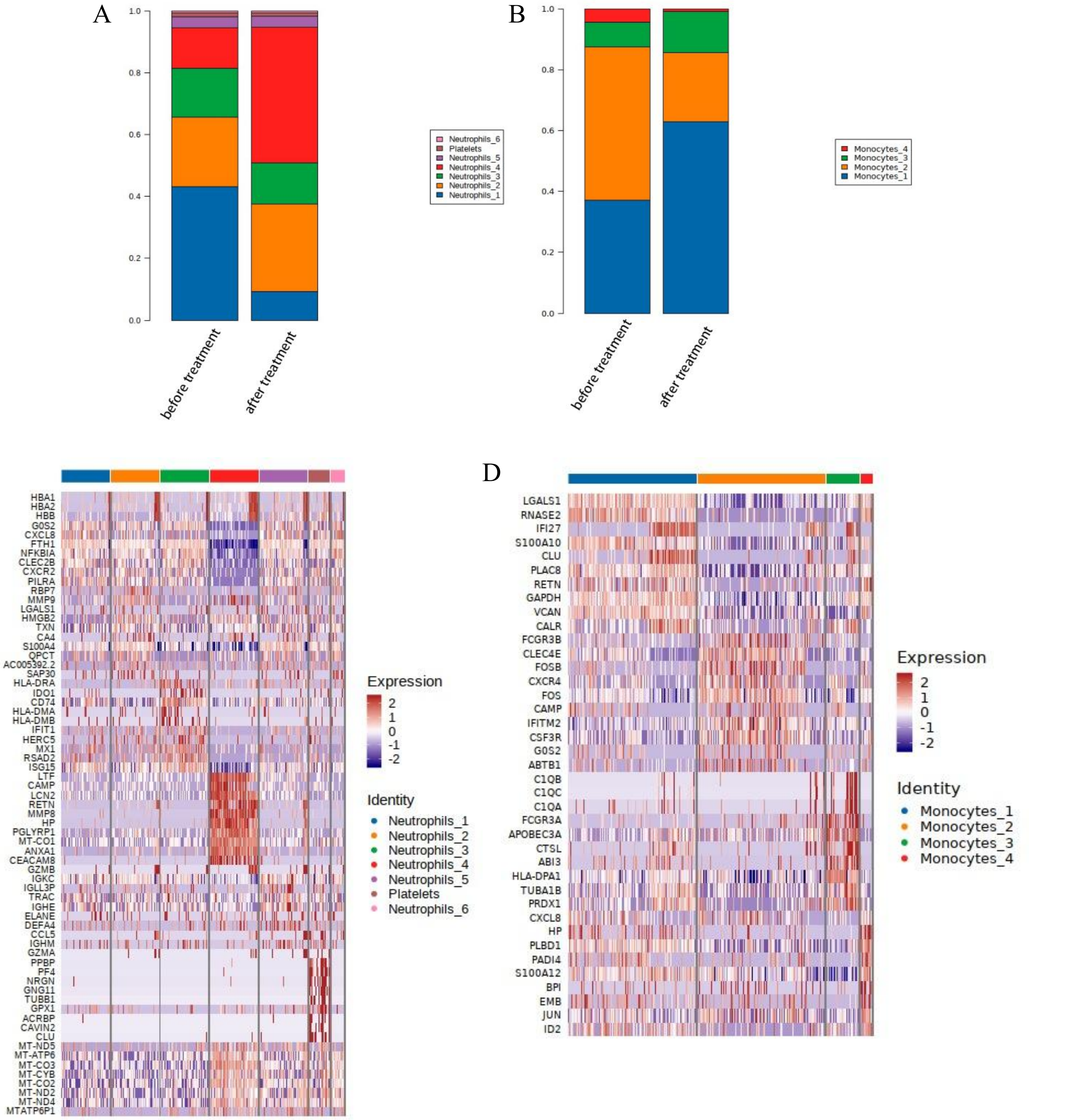

E

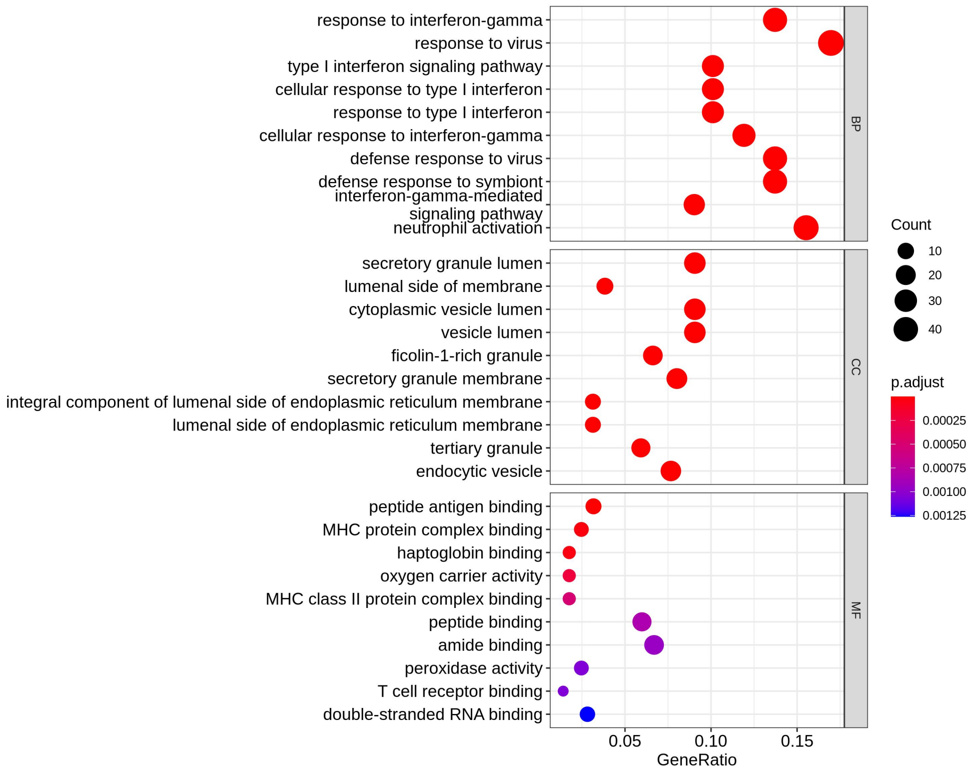

response to interferon-gamma
response to virus
type I interferon signaling pathway
cellular response to type I interferon
response to type I interferon
cellular response to interferon-gamma
defense response to virus
defense response to symbiont
interferon-gamma-mediated signaling pathway
neutrophil activation
BP
secretory granule lumen
lumenal side of membrane
cytoplasmic vesicle lumen
vesicle lumen
ficolin-1-rich granule
secretory granule membrane
integral component of lumenal side of endoplasmic reticulum membrane
lumenal side of endoplasmic reticulum membrane
tertiary granule
endocytic vesicle
CC
peptide antigen binding
MHC protein complex binding
haptoglobin binding
oxygen carrier activity
MHC class II protein complex binding
peptide binding
amide binding
peroxidase activity
T cell receptor binding
double-stranded RNA binding
MF
0.05
0.10
0.15
GeneRatio
Count
10
20
30
40
p.adjust
0.00025
0.00050
0.00075
0.00100
0.00125

F

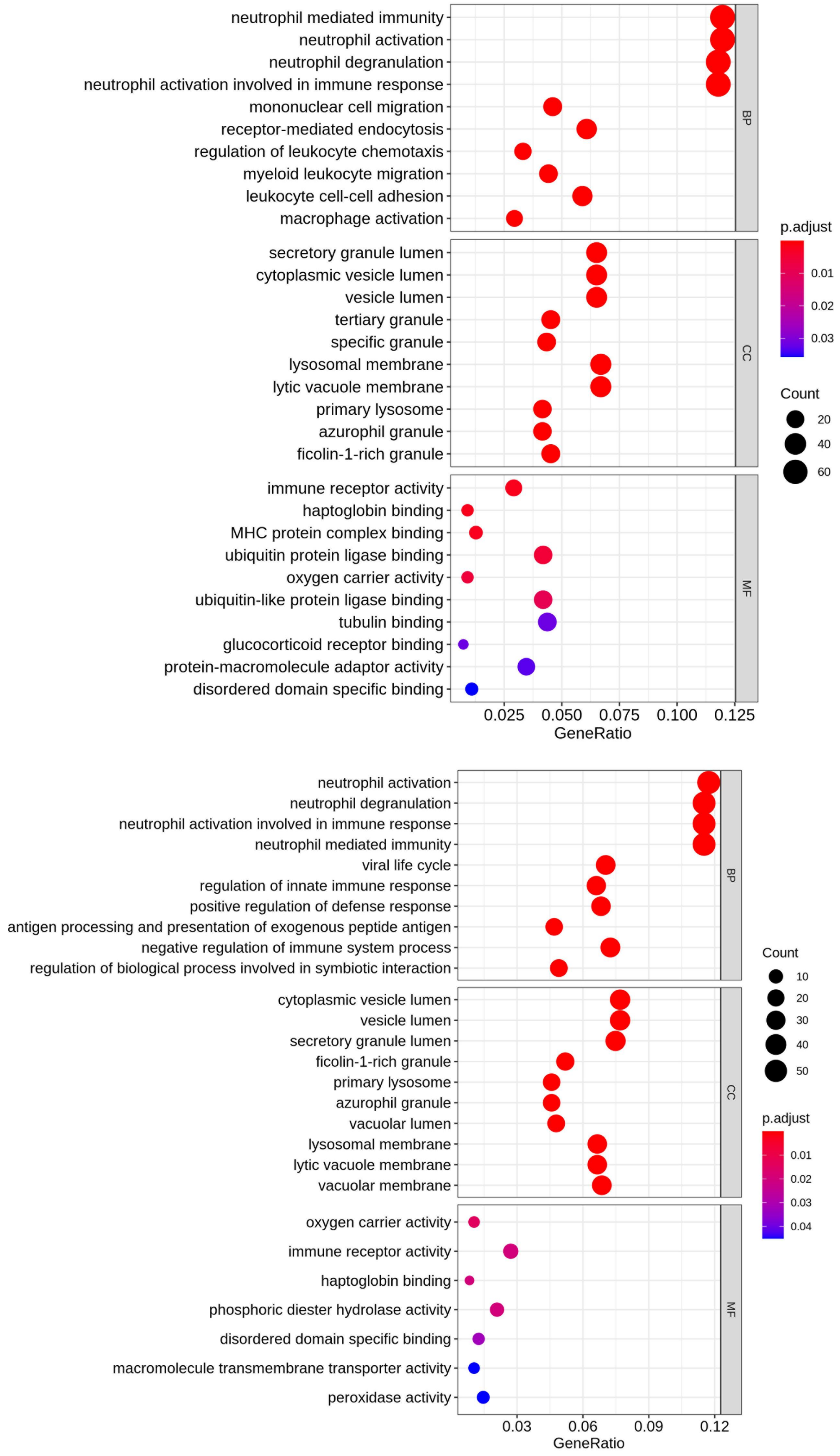
neutrophil mediated immunity
neutrophil activation
neutrophil degranulation
neutrophil activation involved in immune response
mononuclear cell migration
receptor-mediated endocytosis
regulation of leukocyte chemotaxis
myeloid leukocyte migration
leukocyte cell-cell adhesion
macrophage activation
secretory granule lumen
cytoplasmic vesicle lumen
vesicle lumen
tertiary granule
specific granule
lysosomal membrane
lytic vacuole membrane
primary lysosome
azurophil granule
ficolin-1-rich granule
immune receptor activity
haptoglobin binding
MHC protein complex binding
ubiquitin protein ligase binding
oxygen carrier activity
ubiquitin-like protein ligase binding
tubulin binding
glucocorticoid receptor binding
protein-macromolecule adaptor activity
disordered domain specific binding
BP
CC
MF
0.025
0.050
0.075
0.100
0.125
GeneRatio
p.adjust
0.01
0.02
0.03
Count
20
40
60
neutrophil activation
neutrophil degranulation
neutrophil activation involved in immune response
neutrophil mediated immunity
viral life cycle
regulation of innate immune response
positive regulation of defense response
antigen processing and presentation of exogenous peptide antigen
negative regulation of immune system process
regulation of biological process involved in symbiotic interaction
cytoplasmic vesicle lumen
vesicle lumen
secretory granule lumen
ficolin-1-rich granule
primary lysosome
azurophil granule
vacuolar lumen
lysosomal membrane
lytic vacuole membrane
vacuolar membrane
oxygen carrier activity
immune receptor activity
haptoglobin binding
phosphoric diester hydrolase activity
disordered domain specific binding
macromolecule transmembrane transporter activity
peroxidase activity
BP
CC
MF
0.03
0.06
0.09
0.12
GeneRatio
Count
10
20
30
40
50
p.adjust
0.01
0.02
0.03
0.04

Figure S4. (**A**) The fraction of neutrophils was presented before and after PD-1 inhibitor therapy. (**B**) The fraction of monocytes was presented before and after PD-1 inhibitor therapy. (**C**) Top 10 differentially expressed genes that were upregulated in each neutrophil subset were visualized in Heatmap. (**D**) Top 10 differentially expressed genes that were upregulated in each monocyte subset were visualized in Heatmap. (**E**) Top 10 biological processes by GO gene set enrichment analysis in neutrophils_4 were shown in bubble plot according to gene ratio. (**F**) Top 10 biological processes by GO gene set enrichment analysis in monocytes_2 (Up) and monocytes_3 (Down) were shown in bubble plot according to gene ratio.
